# The combined effects of alcohol and marijuana use prior to traumatic brain injury on mortality

**DOI:** 10.1016/j.amsu.2020.11.059

**Published:** 2020-11-27

**Authors:** John J. Leskovan, Puja D. Patel, John Pederson, Aaron Moore, Amer Afaneh, Laura R. Brown

**Affiliations:** a Department of Trauma Surgery, Mercy St. Vincent Medical Center, Toledo, OH, USA; b Superior Medical Experts, Minneapolis, MN, USA; c Department of Surgery, MetroHealth Medical Center, Cleveland, OH, USA

**Keywords:** Alcohol consumption, Marijuana, Cannabis, Brain injuries, Traumatic, Logistic models

## Abstract

**Background:**

Alcohol (ETOH) intoxication is a common comorbidity in traumatic brain injury (TBI), and marijuana (THC) has been implicated as a major risk factor for trauma. The objective this study was to investigate the combined effects of ETOH and THC on mortality after TBI.

**Materials and methods:**

A retrospective review of patient data was performed to assess adult (>18 years) patients with brain injuries between January 2012 and December 2018. Included patients sustained TBI (Abbreviated Injury Scale (AIS 1-6)) and were divided into two groups: No Substances and THC + ETOH.

**Results:**

1085 (median age 52 years [range: 18–97 years]; 33.5% female (364/1085)) patients met the inclusion criteria. Significant differences for mortality at discharge were found between groups (p = 0.0025) with higher mortality in the No Substances group. On multiple logistic regression, a positive test for both ETOH + THC was found not to independently predict mortality at discharge, while age, Glasgow Coma Scale, intensive care unit stay, Injury Severity Score, length of hospital stay, and days on ventilator were independent predictors.

**Conclusions:**

After controlling for confounding variables, positive ETOH + THC screens were not found to be independent predictors of mortality at discharge. Therefore, our results indicated no survival benefit for TBI patients with concomitant ETOH and THC use prior to injury.

## Highlights

⁃ No survival benefit of alcohol and marijuana for all traumatic brain injury levels
⁃ Glasgow Coma Scale predicts mortality for all traumatic brain injury levels
⁃ Length of stay predicts mortality for all traumatic brain injury levels

## 1 Introduction

Alcohol intoxication is a common comorbidity in traumatic brain injury (TBI), with 30%–50% of all TBIs occurring under the influence of alcohol [1,2]. Preclinical studies have indicated that ethanol pretreatment results in a faster recovery with better outcomes after TBI [1,3]. However, numerous clinical studies have examined the relationship of alcohol exposure and risk of mortality in patients with TBI with inconsistent results: some studies have found a positive blood alcohol content (BAC) had no significant relationship with mortality [4,5], while others have found that mortality rate due to TBI with alcohol intoxication is lower compared to those without alcohol intoxication [[6], [7], [8], [9], [10]]. Additionally, marijuana has been implicated as a major risk factor for all types of trauma [1,3]. The anti-inflammatory properties of endocannabinoids have been demonstrated to provide neuroprotective effects after TBI [[11], [12], [13]]. A previous study found a positive tetrahydrocannabinol (THC) screen to be independently associated with survival after TBI [14].

While the risk of injury from alcohol, marijuana, and other drugs in combination is increased [15], the neuroprotective effects of combined marijuana and alcohol have not yet been studied. Few studies have determined the effects of combined drug use on mortality after TBI, and the relationship of combined alcohol and THC on TBI outcomes remains unknown. We hypothesize that the combined effects of marijuana and alcohol will be protective for patients with TBI. The aim of this study is to use a dataset of regional data from 26 regional hospitals to evaluate the combined effects of a positive THC and alcohol screen on patient outcomes after sustaining mild, moderate, and severe traumatic brain injury.

## 2 Materials and Methods

### 2.1 Datasets

Institutional Review Board approval was obtained to analyze the Northern Ohio Regional Trauma Registry. De-identified data was obtained from January 1, 2012 and December 31, 2018 and screened for patients using the following inclusion criteria: TBI (Head Abbreviated Injury Scale (AIS) 1-6), age >18 years, had an alcohol and toxicology screen with documented results, and reported outcome at discharge. Exclusion criteria included: Pediatric (age <18) patients, undocumented toxicology screen or results, and unreported outcomes at discharge.

Included patients were then divided into two groups: 1) No Substances – patients with negative alcohol and toxicology tests and 2) THC + ETOH – patients with positive toxicology for THC and positive blood alcohol content (BAC).

### 2.2 Study variables

Patient data included age, gender, ethnicity, Glasgow Coma Scale (GCS), Injury Severity Score (ISS), complications, and mechanism of injury. Outcome variables included ventilator days, days in intensive care unit (ICU), length of hospital stay (LOS, days), mortality, and discharge disposition.

### 2.3 Statistical analyses

Statistical analyses included Fisher's exact test for comparisons of dichotomous data between groups [11,14]. Odds ratios and 95% CIs were also computed using the Woolf logit method. The Mann-Whitney *U* test was used to compare mean ranks of background characteristics and outcomes between groups. Spearman's rank correlation was used for correlations between background characteristics [16]. Multiple logistic regression with multiple imputation using chained equations and including age, GCS, ICU days, ISS, LOS days, and ventilator days variables was used to identify predictors of discharge mortality rates [17]. Odds ratios were also computed to aid in interpretation of significant outcomes. P-values from logistic regression are computed via Wald's test [18]. In all cases, p-values ≤0.05 were considered significant. Statistics were performed in RStudio (Version 1.2.5033).

## 3 Results

Cumulatively, 1085 patients were included in this analysis. For each group of alcohol and toxicology test results, the number of patients in each group included 909 (83.8%) No Substances and 176 (16.2%) THC + ETOH (Table 1).

**Table 1:** Patient characteristics by drug class.

Characteristic	No Substances [n = 909]	THC + ETOH [n = 176]
**Age, mean (SD)**	54.68 (±21.28)	37.88 (±13.06)
**Female, n (%)**	333 (36.63%)	31 (17.61%)
**GCS, median (IQR)**	15 (13–15)	15 (7–15)
**ISS, median (IQR)**	12 (6–21)	9 (5–17)
**ICU days, median (IQR)**	1 (0–3)	1 (0–3)
**LOS days, median (IQR)**	3 (1–7)	2 (1–5.25)
**Ventilator days, median (IQR)**	0 (0–2)	1 (0–3)
**Complications (n)**	192	43

Data are mean ± SD, n (%), or median (IQR); GCS=Glasgow Coma Scale; ICU=Intensive Care Unit; ISS=Injury Severity Score; LOS=Length of stay; THC=tetrahydrocannabinol.

Dichotomous comparisons between sex and mortality at discharge between groups were performed (Table 2). Significant differences were found for sex between the THC + ETOH and No Substances groups (OR 2.602 [95% CI: 1.733 to 3.905], p < 0.001) with more females in the No Substances group. Significant differences for mortality at discharge were also found between THC + ETOH and No Substances groups (OR 3.528 [95% CI: 1.410 to 8.825], p = 0.0025) with higher mortality in the No Substances group.

**Table 2:** Dichotomous comparisons of sex and mortality by group.

Sex						
	F	M	Total	Odds Ratio	95% CI	P value
**No Substances**	333	576	909	2.60	1.73 to 3.91	<0.001
**ETOH + THC**	32	144	176			
**Mortality at Discharge**						
**No Substances**	85	824	909	3.53	1.41 to 8.83	0.0025
**ETOH + THC**	5	171	21			

THC=tetrahydrocannabinol; CI=Confidence Interval.

Comparisons of ranked data between groups include age, LOS (days), ICU stay (days), ventilator (days), GCS, ISS, and number of complications (Table 3). Significant differences in LOS were found between THC + ETOH and No Substances (p < 0.001) groups with longer LOS in the No Substances group. Additionally, THC + ETOH group had significantly higher GCS scores than the No Substances (p = 0.005) group. No significant differences were found between any of the groups for age, ICU days, number of complications, ISS, and ventilator days [Table 3].

**Table 3:** Comparisons of ranked data by group.

	Median	Difference	P-Value
Age			
ETOH + THC	57.0 [n = 909]	−23.0	>0.999
No Substances	34.0 [n = 176]		
**Length of Stay (days)**			
ETOH + THC	3.0 [n = 909]	−1.0	<0.001
No Substances	2.0 [n = 176]		
**ICU (days)**			
ETOH + THC	1.0 [n = 822]	0.0	0.875
No Substances	1.0 [n = 153]		
**Ventilator (days)**			
ETOH + THC	0.0 [533]	1.0	0.081
No Substances	1.0 [n = 113]		
**GCS**			
ETOH + THC	15.0 [n = 796]	0.0	0.005
No Substances	15.0 [144]		
**ISS**			
ETOH + THC	12.0 [n = 906]	−3.0	0.055
No Substances	9.0 [n = 176]		
**Complications**			
ETOH + THC	1.0 [n = 192]	0.0	0.844
No Substances	1.0 [n = 40]		

THC=tetrahydrocannabinol; CI=Confidence Interval.

### 3.1 Multiple logistic regression

**Fig. 1:**
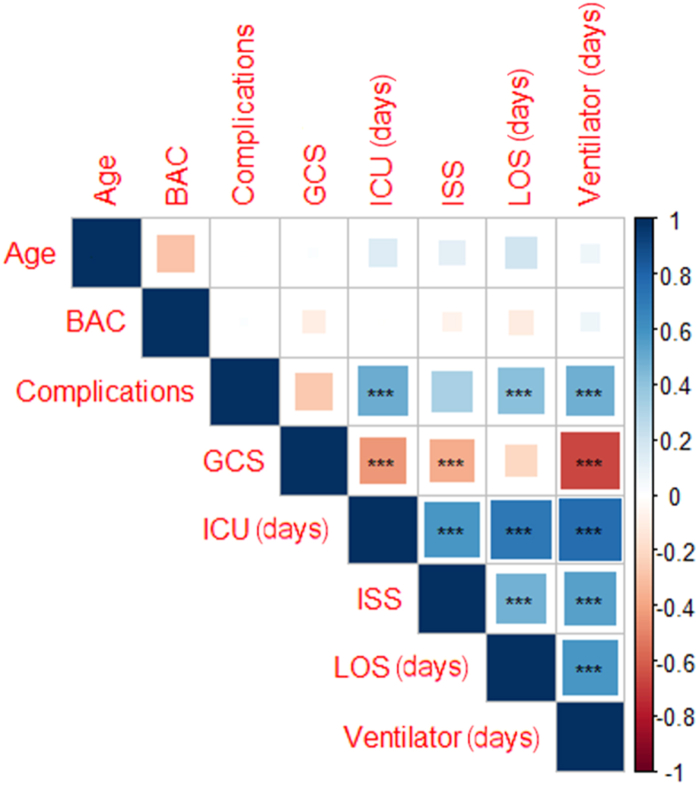
**Correlation Matrix using Spearman's rank correlation.** Blue represents positive correlations, and red symbolizes inverse correlations. *p < 0.05, **p < 0.01, ***p < 0.001. GCS=Glasgow Coma Scale 2; ISS=Injury Severity Score; LOS=Length of stay; ICU=Intensive Care Unit. Complications vs. ICU (days) (r = 0.494) Complications vs. LOS (days) (r = 0.415), Complications vs. Ventilator.days (r = 0.483), GCS vs. ICU (days) (r = −0.433), GCS vs. ISS = (r = −0.367), GCS vs. Ventilator.days (r = −0.664), ICU.days vs. ISS (r = 0.582), ICU.days vs. LOS.days (r = 0.716), ICU.days vs. Ventilator.days (r = 0.761), ISS vs. LOS.days (r = 0.474), ISS vs. Ventilator.days (r = 0.544), LOS.days vs. Ventilator.days (r = 0.581). (For interpretation of the references to colour in this figure legend, the reader is referred to the Web version of this article.)

**Fig. 2:**
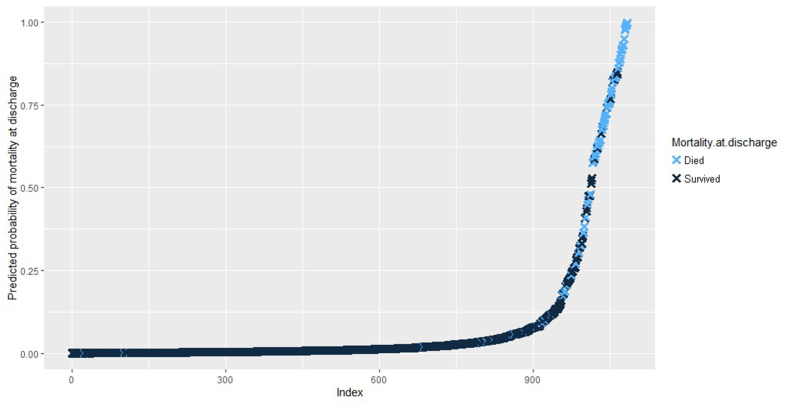
**Results of multiple logistic regression from regressing background characteristics on mortality at discharge.** Light blue data points represent actual patients that died at discharge and dark blue data points represent patients that did not survive past 90 days. The y-axis shows the predicted probability of mortality at discharge for individual patients using a predictive model obtained from multiple logistic regression with multiple imputation using chained equations. From the equation, McFadden's pseudo r2 was 0.535 (p < 0.001), showing that the model can reliably predict mortality at discharge at the α = 0.05 level. (For interpretation of the references to colour in this figure legend, the reader is referred to the Web version of this article.)

On multiple logistic regression, the following variables were identified as independent predictors of mortality at discharge: Age (OR: 1.043 [95% CI: 1.023, 1.065], p < 0.001), GCS (OR: 0.769 [95% CI: 0.717, 0.820], p < 0.001), ICU days (OR = 1.482 [95% CI: 1.173, 1.902], p = 0.001), ISS (OR: 1.089 [95% CI: 1.059, 1.120], p < 0.001), and LOS days (OR: 0.584 [95% CI: 0.480, 0.692], p < 0.001). BAC, cause of TBI, drug class, race, and sex were not significant predictors of mortality at discharge. A correlation matrix using Spearman's rank correlation is shown in Fig. 1. Results of the multiple logistic regression from regressing background characteristics on mortality at discharge is displayed in Fig. 2, where McFaddon's Pseudo R^2^ of the regression model was 0.535 (p < 0.001).

## 4 Discussion

Our results demonstrate TBI patients with a positive toxicology for THC and alcohol were found to have significantly lower mortality at discharge when compared to patients with no substances (negative toxicology). However, in a multiple logistic regression, combined BAC and drug class were not found to be independent predictors of mortality at discharge, while age, GCS, ICU days, ISS, and LOS were found to be independent predictors of mortality.

Though somewhat contested, the effect of alcohol intoxication on patients with TBI has been shown in many studies to improve mortality [[6], [7], [8],^10^]. A meta-analysis of observation studies by Raj et al. included 11 studies with 95,941 patients, and found that positive BAC was significantly associated with lower mortality rates in moderate to severe TBI [9]. Conversely, a meta-analysis examining the impact of day-of-injury alcohol consumption on outcomes after TBI by Mathias et al., found that positive blood alcohol levels were associated with significantly poorer cognitive outcomes and higher levels of disability. Overall, they found that day-of-injury alcohol consumption is not consistently associated with better or worse outcomes, other than subtle cognitive deficits [7].

The effect of marijuana on TBI is far less studied than alcohol, though many preclinical studies have shown THC is associated with neuroprotective effects including alleviation of brain edema, attenuated cell apoptosis, improved neurobehavioral function, and enhanced cerebral blood flow [[11], [12]]. These effects are partially attributed to the upregulation of NFE-2 factor, which regulates the cellular antioxidant response, following TBI and modulation of the mitochondrial apoptotic pathway [[12], [13]]. A study by Nguyen et al. found that after adjusting for differences between study cohorts, a positive THC screen was found to be associated with increased survival after TBI [14].

With the individual effects of alcohol and marijuana on TBI still contested, their combined effects on mortality have not been explicitly studied. DiGiorgio et al. investigated the impact of drug and alcohol intoxication on GCS assessment in patients with TBI, and found that intoxicating substances can confound GCS score with impaired patients having a significantly higher mean change in GCS score compared with patients with a negative screening test [19]. A retrospective review by O'Phelan et al. studied the impact of substance abuse on mortality in patients with TBI by comparing amphetamine, benzodiazepine, narcotic, cannabis, cocaine, alcohol, polydrug, and polydrug, excluding alcohol, and found that methamphetamine use was a significant predictor of mortality [20]. They also demonstrated that patients who tested positive for methamphetamine were also more likely to test positive for cannabis and hypothesized the synergistic effects of methamphetamine and THC may have contributed to overall lower mortality in this cohort [20]. In our study we employed a logistic regression model that controlled for age, gender, GCS, ICU days, LOS days, ventilator days, ISS, and complications and found neither THC nor a positive BAC screen to be independent predictors of mortality, which is consistent with the analysis by O'Phelan et al.

### 4.1 Limitations

Several limitations of this study should be noted, primarily that it was retrospective in nature, and some data was limited on these patients, including clinical course and surgical treatment. Additionally, past drug history was not collected, which made it impossible to distinguish between chronic and acute drug use. Limitations in toxicology screens may have given positive THC screening results even for patients who had not been actively intoxicated or recently used before TBI, if they had used THC in the recent past (4.6–15.4 days) [21]. Mortality at discharge was used as the end point, which did not consider the long-term effects of BAC after TBI, and BAC levels were not quantified in our analyses.

## 5 Conclusions

In our study, we found that after TBI, a combined positive THC and BAC screen was not an independent predictor of mortality at discharge when controlling for confounding variables. While the neuroprotective and anti-inflammatory effects of both THC and alcohol in the setting of TBI have been demonstrated in preclinical and clinical studies, their interaction has not been studied in the context of TBI, and further research is needed to investigate their combined effects on mortality and to develop treatment guidelines for this patient population.

## Funding

This research did not receive any specific grant from funding agencies in the public, commercial, or not-for-profit sectors.

## Ethical statement

Institutional Review Board approval was obtained to analyze deidentified patient data.

## Author contribution

John J. Leskovan: Conceptualization, Supervision, Methodology, Writing - review & editing, Puja D. Patel: Writing - original draft, Writing - review & editing, John Pederson: Methodology, Investigation, Formal analysis, Data curation, Writing - review & editing, Visualization, Aaron Moore: Writing - review & editing, Amer Afaneh: Writing - review & editing, Laura R. Brown: Writing - review & editing

## Annals of medicine and surgery

The following information is required for submission. Please note that failure to respond to these questions/statements will mean your submission will be returned. If you have nothing to declare in any of these categories then this should be stated.

PDP and JP contract with Superior Medical Experts. The remaining authors report no conflict of interest.

All authors must disclose any financial and personal relationships with other people or organisations that could inappropriately influence (bias) their work. Examples of potential conflicts of interest include employment, consultancies, stock ownership, honoraria, paid expert testimony, patent applications/registrations, and grants or other funding.

The authors declare no funding supported this work.

All sources of funding should be declared as an acknowledgement at the end of the text. Authors should declare the role of study sponsors, if any, in the collection, analysis and interpretation of data; in the writing of the manuscript; and in the decision to submit the manuscript for publication. If the study sponsors had no such involvement, the authors should so state.

## Ethical approval

Research studies involving patients require ethical approval. Please state whether approval has been given, name the relevant ethics committee and the state the reference number for their judgement.

Institutional Review Board approval was obtained to analyze deidentified patient data.

## Consent

Studies on patients or volunteers require ethics committee approval and fully informed written consent which should be documented in the paper.

Authors must obtain written and signed consent to publish a case report from the patient (or, where applicable, the patient's guardian or next of kin) prior to submission. We ask Authors to confirm as part of the submission process that such consent has been obtained, and the manuscript must include a statement to this effect in a consent section at the end of the manuscript, as follows: "Written informed consent was obtained from the patient for publication of this case report and accompanying images. A copy of the written consent is available for review by the Editor-in-Chief of this journal on request”.

Patients have a right to privacy. Patients’ and volunteers' names, initials, or hospital numbers should not be used. Images of patients or volunteers should not be used unless the information is essential for scientific purposes and explicit permission has been given as part of the consent. If such consent is made subject to any conditions, the Editor in Chief must be made aware of all such conditions.

Even where consent has been given, identifying details should be omitted if they are not essential. If identifying characteristics are altered to protect anonymity, such as in genetic pedigrees, authors should provide assurance that alterations do not distort scientific meaning and editors should so note.

## Registration of research studies

In accordance with the Declaration of Helsinki 2013, all research involving human participants has to be registered in a publicly accessible database. Please enter the name of the registry and the unique identifying number (UIN) of your study.

You can register any type of research at http://www.researchregistry.com to obtain your UIN if you have not already registered. This is mandatory for human studies only. Trials and certain observational research can also be registered elsewhere such as: ClinicalTrials.gov or ISRCTN or numerous other registries.

1. Name of the registry: Research Registry.

2. Unique Identifying number or registration ID: researchregistry6071.

3. Hyperlink to your specific registration (must be publicly accessible and will be checked): https://www.researchregistry.com/register-now#home/registrationdetails/5f75d4e2366ed2001552f475/

## Guarantor

John J Leskovan, DO, FACS, FACOS

Department of Trauma Surgery

Mercy St. Vincent Medical Center

2213 Cherry St, Toledo OH, 43608

Email: jjleskovan@mercy.com

Telephone: 419-251-4674

Fax: 419-251-3862

## Declaration of competing interest

PDP and JP contract with Superior Medical Experts. The authors report no conflict of interest concerning the materials or methods used in this manuscript.
